# Usual blood pressure, atrial fibrillation and vascular risk: evidence from 4.3 million adults

**DOI:** 10.1093/ije/dyw053

**Published:** 2016-04-28

**Authors:** Connor A Emdin, Simon G Anderson, Gholamreza Salimi-Khorshidi, Mark Woodward, Stephen MacMahon, Terrence Dwyer, Kazem Rahimi

**Affiliations:** 1 George Institute for Global Health, University of Oxford, Oxford, UK; 2 Cardiovascular Research Group, Institute of Cardiovascular Sciences, University of Manchester, Manchester, NH, USA; 3 George Institute for Global Health, University of Sydney, Sydney, NSW, Australia; 4 Department of Epidemiology, Johns Hopkins University, Baltimore,MD, USA; 5 Division of Cardiovascular Medicine, Radcliffe Department of Medicine, University of Oxford, Oxford, UK

**Keywords:** Atrial fibrillation, blood pressure, epidemiology, cardiovascular disease

## Abstract

**Background:** Although elevated blood pressure is associated with an increased risk of atrial fibrillation (AF), it is unclear if this association varies by individual characteristics. Furthermore, the associations between AF and a range of different vascular events are yet to be reliably quantified.

**Methods:** Using linked electronic health records, we examined the time to first diagnosis of AF and time to first diagnosis of nine vascular events in a cohort of 4.3 million adults, aged 30 to 90 years, in the UK.

**Results**: A 20-mmHg higher usual systolic blood pressure was associated with a higher risk of AF [hazard ratio (HR) 1.21, 95% confidence interval (CI) 1.19, 1.22]. The strength of the association declined with increasing age, from an HR of 1.91 (CI 1.75, 2.09) at age 30-40 to an HR of 1.01 (CI 0.97, 1.04) at age 80-90 years. AF without antithrombotic use at baseline was associated with a greater risk of any vascular event than AF with antithrombotic usage (*P* interaction < 0.0001). AF without baseline antithrombotic usage was associated with an increased risk of ischaemic heart disease (HR 2.52, CI 2.23, 2.84), heart failure (HR 3.80, CI 3.50, 4.12), ischaemic stroke (HR 2.72, CI 2.19, 3.38), unspecified stroke (HR 2.59, CI 2.25, 2.99), haemorrhagic stroke, chronic kidney disease, peripheral arterial disease and vascular dementia, but not aortic aneurysm.

**Conclusions:** The association between elevated blood pressure and AF attenuates with increasing age. AF without antithrombotic usage is associated with an increased risk of eight vascular events.

## Introduction

Atrial fibrillation (AF) is the most common cardiac arrhythmia and poses an increasing burden worldwide.^1^^,^^2^ AF was associated with five times the risk of stroke in analyses of the Framingham cohort,^3^^,^^4^ and is also associated with a reduced quality of life.^5^ Despite broader usage of anticoagulant therapy for prevention of stroke,^6^ the condition remains associated with an increased risk of death.^7^

Elevated blood pressure (BP) is a risk factor for incident AF. In an analysis of 4731 individuals free of AF, hypertension, defined as provision of antihypertensive medication or a systolic blood pressure of 160 mmHg or greater, was associated with a 50% higher risk of incident atrial fibrillation in men and a 40% higher risk in women.^8^ However, it is unclear if the relationship between blood pressure and AF is consistent among various subpopulations. Previous cohort studies (Supplementary Table 1, available as Supplementary data at *IJE* online) have not been sufficiently large to allow reliable investigation of the relationship between BP and AF by important patient characteristics.

The relationship between AF and vascular risk is also unclear. Previous cohort studies have consistently recognized atrial fibrillation as a risk factor for stroke and heart failure. However, they have provided conflicting results on the existence and strength of the relationship between atrial fibrillation and coronary heart disease, peripheral arterial disease, chronic kidney disease and other vascular disease (Supplementary Table 1). Reliable estimation of atrial fibrillation as a risk factor for such vascular events, in a contemporary setting, may allow for better understanding of risks and stratification of management strategies.

To clarify these existing uncertainties, we sought to reliably determine the association between blood pressure and atrial fibrillation and to further reliably determine the association between atrial fibrillation and nine different vascular events, using data from a large contemporary cohort.

## Methods

### Participants and exposures

We used the Clinical Practice Research Datalink (CPRD), a primary care database previously validated for epidemiological research.^9^^,^^10^ CPRD has been demonstrated to be nationally representative of the UK population in terms of age, sex and ethnicity;^11^^,^^12^ 87% of participants in CPRD are White, 6% are South Asian, 3.7% are Black and 3.7% are other or mixed, highly similar to the UK census.^12^ Eligible participants were also linked to Hospital Episode Statistics (secondary care/hospitalization data) and Office of National Statistics Data (cause-specific mortality data). We included all participants who were registered at a research standard general practice for at least 1 year and had at least one BP measurement between 1990 and 2013.We used this first BP measurement at a general practice as our exposure. For other covariates, specifically sex, age, body mass index (BMI), smoking status, total cholesterol and high-density lipoprotein (HDL) cholesterol, we utilized the most recent measurement within 2 years of the baseline BP measurement. If a measurement was not available within 2 years of the baseline BP measurement, we classified it as missing. We also classified individuals as having diabetes at baseline, if they had been diagnosed with diabetes or prescribed antidiabetic drugs before their baseline BP measurement. We similarly classified individuals as having AF at baseline, if they had been diagnosed with AF (in either primary care or secondary care) prior to the baseline blood pressure measurement. We excluded all individuals with a previous diagnosis of vascular disease (ischaemic heart disease, stroke, heart failure, peripheral arterial disease, chronic kidney disease or vascular dementia) to minimize the risk of confounding and reverse causality.

Patients were defined as having baseline antihypertensive or lipid lowering therapy if they were prescribed an antihypertensive or lipid-lowering drug in the 2 years preceding a baseline BP measurement. Patients were defined as using anticoagulant therapy or antiplatelet therapy at baseline, if they were prescribed an anticoagulant or antiplatelet agent within 3 years of the baseline BP measurement.

### Endpoints

For the analysis of the association between blood pressure and AF, our primary endpoint was incidence of AF (ICD-10 code I48), in either primary care, secondary care (hospitalization) or death. Previous research has demonstrated AF diagnoses in CPRD to be highly reliable^13^ and to have similar associations with risk factors as traditional cohort studies.^14^ Previous research has also demonstrated endpoints in CPRD to have high specificity.^15^ Participants were censored at the earliest of an occurrence of either a presentation of AF, or transfer out of practice, or death or last collection date of practice.

For the analysis of the further association between AF and vascular risk, our primary endpoint was incidence of nine vascular events: ischaemic stroke, haemorrhagic stroke, stroke unspecified, ischaemic heart disease (a composite of fatal ischaemic heart disease and non-fatal myocardial infarction), heart failure, chronic kidney disease, peripheral arterial disease, aortic aneurysm and vascular dementia. We examined both fatal vascular events and combined fatal and non-fatal vascular events. Definitions of events are provided (Supplementary Table 2, available as Supplementary data at *IJE* online).Participants were censored as above.

### Statistical analysis

To examine blood pressure as a risk factor for incident AF, we excluded all individuals who were diagnosed with atrial fibrillation before the baseline blood pressure measurement. Cox proportional hazard models, stratified by practice, were used with blood pressure taken both as a continuous variable [per 20 mmHg/10 mmHg higher systolic blood pressure (SBP) / diastolic blood pressure (DBP)] and categorical variable (in 10-mmHg groups for SBP). We estimated 95% confidence intervals (CIs) for categorical blood pressure using floating absolute risks.^16^ The primary model was adjusted for age, sex, BMI, smoking status and diabetes. Rather than adjusting for antihypertensive usage in this model, we excluded individuals on BP-lowering medication in a sensitivity analysis (described below), as previous research has suggested that antihypertensive usage may modify the association between BP and risk of cardiovascular disease, even after adjustment.^10^ The proportional hazards assumption was verified using Schoenfeld residuals.

To examine whether AF was a risk factor for vascular events, we similarly used stratified Cox models. We examined whether there was an interaction between baseline atrial fibrillation and baseline usage of antithrombotic therapy (either anticoagulant therapy or antiplatelet therapy).^17^^,^^18^ The primary model was adjusted for sex, age, BMI, smoking status, diabetes, baseline anticoagulant usage, baseline antiplatelet usage, baseline antihypertensive usage and baseline lipid-lowering usage. We included usage of anticoagulant therapy, antihypertensive therapy and lipid-lowering therapy in this model as these medications may modify the risk of vascular disease associated with AF.^19–22^

As previously described,^23^^,^^24^ we used methods similar to the Emerging Risk Factors Collaboration (ERFC) to adjust for regression dilution bias.^25–28^ That is, we regressed serial blood pressure measurements within the median follow-up [available for 3 248 391 (76%) participants] on the baseline blood pressure measurement, but used generalized estimating equations, rather than linear models, to account for multiple serial blood pressure measurements among participants. A mean of 7.4 BP measurements during follow-up were available for each participant, which increased with increasing age (mean 5.1 measurements between ages 30 and 39 vs mean 8.9 measurements between ages 80 and90). Regression dilution ratios were calculated as the inverse of the coefficient relating the serial measurements to the baseline measurement. Regression dilution ratios of 2.2 for systolic blood pressure (SBP) and 2.6 for diastolic blood pressure (DBP) were estimated. Continuous hazard ratios for measured blood pressure (i.e. per 20/10 mm Hg) were then multiplied by these ratios, to estimate the association for usual blood pressure. For example, if a hazard ratio for baseline systolic blood pressure of 1.5 was calculated, the hazard ratio for usual systolic blood pressure was calculated as exp(2.2*log(1.5)) = 2.5. For displaying hazard ratios of blood pressure as a categorical variable (i.e. 120-130 mm Hg measured SBP), measured blood pressure was ‘shrunk’ towards the overall mean blood pressure by the calculated regression dilution ratios, as performed previously.^29^ For example, if the overall mean of the baseline SBP measurements was 130 mmHg and the mean of a specific blood pressure category was 140, the mean usual blood pressure of that category was calculated as [(140 mmHg-130 mmHg)/2.2] + 130 = 135 mmHg.

Multiple imputation using chained equations was used to impute missing covariates; five imputations were generated.

### Sensitivity analyses

In our assessment of the association of usual blood pressure with risk of AF, we conducted 10 sensitivity analyses. First, we adjusted for total cholesterol and HDL cholesterol. Second, we further adjusted for the period of the baseline BP measurement (approximately 5-year groups). Third and fourth, we excluded the first 2 years and 4 years of follow up, to examine the possibility that our results were influenced by reverse causality (e.g. undiagnosed heart failure causing AF).Fifth, for the analysis of the association between BP and AF, we excluded individuals prescribed BP-lowering drugs at baseline. Sixth, from the analysis of the association between AF and vascular risk, we excluded all individuals who were diagnosed with AF more than 3 years before baseline BP measurement, to examine whether our results were influenced by individuals who had a temporary diagnosis in the past but did not have a recent diagnosis of AF at baseline. Seventh, we excluded all individuals who developed new-onset AF during follow-up, but did not have AF at baseline. Eighth, we only analysed individuals who were diagnosed through primary care with the read code ‘G573000 Atrial fibrillation’, excluding individuals with non-specific diagnoses of atrial fibrillation to exclude cases of atrial flutter or potential postoperative atrial fibrillation. Finally 9th and 10th, for the analysis of the association between AF and vascular risk, we excluded individuals prescribed antiplatelet therapy and those prescribed anticoagulant therapy, respectively, to examine whether associations of AF with vascular risk varied by baseline usage of antiplatelet therapy vs anticoagulant therapy.

Analyses were conducted using R version 3.0.

## Results

A total of 4 301 349 individuals qualified for our analyses (Supplementary Figure 1, available as Supplementary data at *IJE* online); 32 155 (0.7%) individuals had a previous diagnosis of AF and were excluded from the analysis of the association between blood pressure and new-onset atrial fibrillation (Table 1). The median follow-up was 6.9 years (interquartile interval 3.0, 11.2); a further 128 468 (3.0%) individuals developed AF during this period (Table 1). Absolute rates of AF by age and sex are provided (Supplementary Table 3, available as Supplementary data at *IJE* online, ).

**Table 1.** Characteristics of participants included in the analysis of the association between blood pressure and atrial fibrillation

	**≤ 120 mmHg**	**121-140 mmHg**	**> 140 mmHg**	**Overall**
*N*	1 500 753	1 595 134	1 173 307	4 269 194
Age at baseline (IQI)	39 (33, 48)	47 (37, 58)	59 (48, 70)	46 (36, 59)
Women	996 546 (66.4%)	794 752 (49.8%)	582 941 (49.7%)	2 374 239 (55.6%)
BMI (IQI)	24.3 (21.9, 27.4)	26.3 (23.5, 29.7)	27.4 (24.4, 31.1)	25.8 (23.0, 29.3)
Smoking status				
Current smoker	375 663 (30.0%)	367 380 (28.2%)	227 331 (24.8%)	970 374 (28.0%)
Never smoker	683 906 (54.7%)	702 436 (53.9%)	497 242 (54.3%)	1 883 584 (54.3%)
Ex-smoker	190 863 (15.3%)	234 344 (18.0%)	191 047 (20.9%)	616 254 (17.8%)
Cholesterol				
Total (IQI)	5.2 (4.5, 6.0)	5.4 (4.7, 6.2)	5.6 (4.9, 6.4)	5.5 (4.7, 6.2)
HDL (IQI)	1.4 (1.1, 1.7)	1.3 (1.1, 1.6)	1.4 (1.1, 1.6)	1.4 (1.1, 1.6)
Most deprived quintile	281 413 (18.8%)	297 558 (18.7%)	229 012 (19.5%)	807 983 (18.9%)
Antihypertensive at baseline	57 573 (3.8%)	138 674 (8.7%)	234 798 (20.0%)	431 045 (10.1%)
Antihypertensive during follow-up	201 578 (13.4%)	410 998 (25.8%)	623 419 (53.1%)	1 235 995 (29.0%)
Lipid lowering at baseline	14 034 (0.9%)	34 419 (2.2%)	33578 (2.9%)	82 031 (1.9%)
Lipid lowering during follow-up	110 389 (7.4%)	243 492 (15.3%)	311 998 (26.6%)	665 879 (15.6%)
Diabetes at baseline	26 412 (1.8%)	53 484 (3.4%)	57 572 (4.9%)	137 468 (3.2%)

IQI refers to interquartile interval (25th percentile, 75th percentile). Proportion of variables missing: BMI (30.5%), smoking status (18.7%), total cholesterol (73.0%), HDL cholesterol (80.1%).

Individuals with baseline AF were included in the analysis of the association between baseline AF and vascular risk (Supplementary Table 4, available as Supplementary data at *IJE* online). During 31.9 million patient-years of follow-up, 421 084 vascular events were recorded, including 76 121 ischaemic heart disease events, 69 332 stroke events and 66 473 heart failure events. These included 409 511 vascular events among individuals without AF at baseline and 11 573 vascular events among individuals with AF at baseline.

### Association of usual blood pressure with the risk of AF

**Figure 1:**
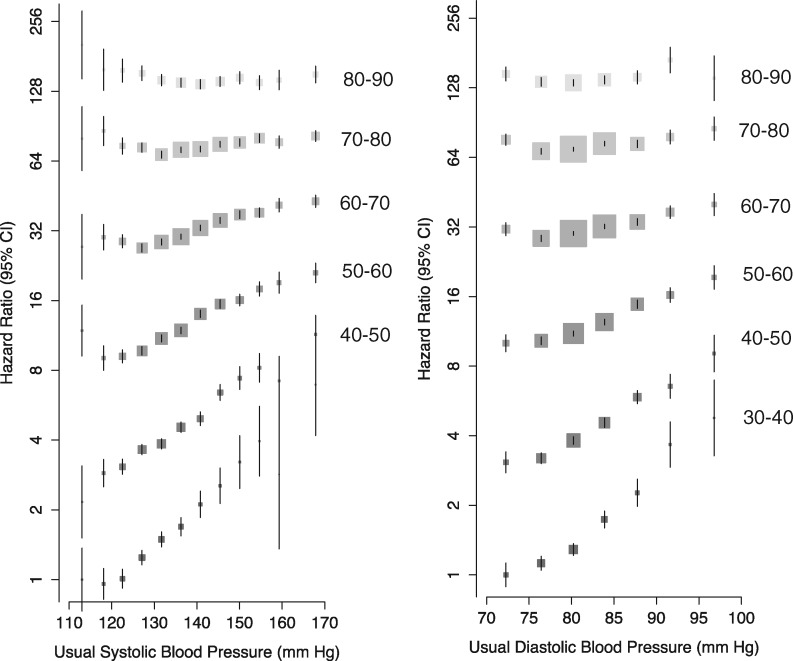
Adjusted hazard ratios of systolic blood pressure and diastolic blood pressure for incident atrial fibrillation by age. Adjustments were for BMI, smoking status, sex, baseline diabetes and the interaction between age as a categorical variable and systolic and diastolic blood pressures as categorical variables, respectively (plotted). Confidence intervals are displayed as floating absolute risks with no reference category. Area of each square is proportional to the inverse variance of the estimate. Hazard ratios for each category are displayed relative to the reference category (individuals aged 30-40 with usual SBP < 115 mmHg). As a result, individuals aged 80-90 are at approximately 128 times the risk of AF compared with individuals aged 30-40 years.

**Figure 2:**
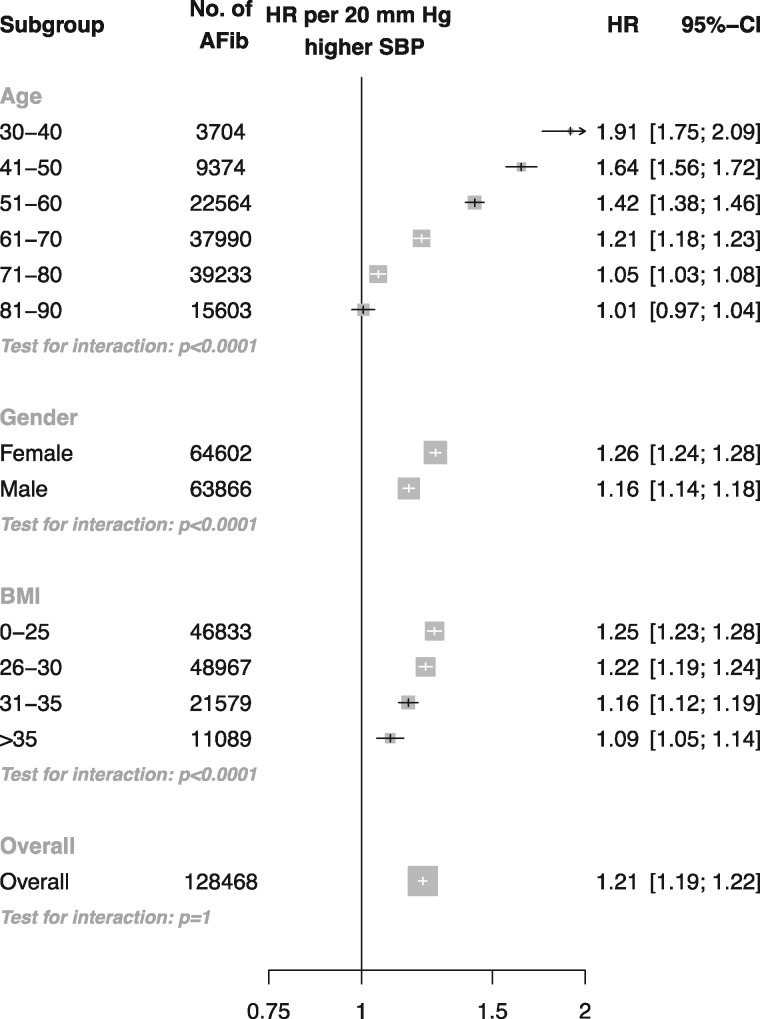
Adjusted hazard ratios of 20 mmHg higher usual SBP for incident atrial fibrillation stratified by patient subgroup. Adjustments were for age, BMI, smoking status, sex and baseline diabetes. For subgroups of age, adjustment was also for age category and the interaction between systolic BP and age category (plotted). For subgroups of sex, adjustment was also for the interaction between sex and systolic BP (plotted). For subgroups of BMI, adjustments were also for BMI category and the interaction between systolic BP and BMI category (plotted). Area of each square is proportional to the inverse variance of the estimate.

Usual SBP was positively related to the risk of AF, with no evidence of a nadir in the age range 30-60 years (Figure 1). At ages 60 to 90 years, SBP was positively related to the risk of AF. However, the association appeared to plateau at approximately 120 mmHg. When analysed as a continuous variable, a 20-mmHg higher SBP was associated with a near doubling in risk of AF at age 30-40 years (HR 1.91, CI 1.75, 2.09). The strength of the association declined with age (Figure 2). However, due to the increasing baseline absolute risk of AF with increasing age, the absolute risk of AF associated with a 20-mmHg higher SBP increased until approximately age 51-60. Risk of AF was greater in women than men per 20 mmHg higher SBP, and there was evidence of decline in the association by BMI (*P* interaction < 0.0001). Overall, per 20 mmHg higher usual SBP, the risk of atrial fibrillation increased by 21% (HR 1.21, CI 1.19, 1.22).

A 10-mmHg higher usual DBP had a similar strength of association with risk of atrial fibrillation compared with a 20-mmHg higher SBP (HR 1.21, CI 1.19, 1.23, Supplementary Figure 2, available as Supplementary data at *IJE* online). However, unlike SBP, a 10-mmHg higher DBP was still positively associated with the risk of AF at ages 81-90 years (CI 1.06, HR 1.02, 1.11) despite a declining HR with increasing age.

None of the sensitivity analyses provided inferences that conflicted with our main results (Supplementary Figures 3-8, available as Supplementary data at *IJE* online).

### Association of AF with incident vascular disease

**Figure 3:**
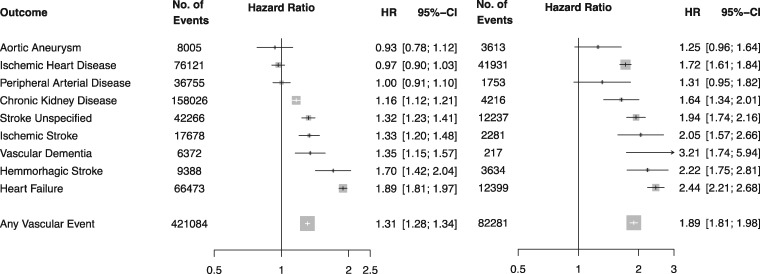
Adjusted hazard ratios of baseline atrial fibrillation for nine different vascular events. Adjustments were for age, BMI, smoking status, sex, baseline diabetes, baseline antihypertensive use, baseline lipid-lowering drug (statin) use, baseline anticoagulant usage, baseline antiplatelet usage and baseline atrial fibrillation (plotted). Restricted to (A) fatal and non-fatal vascular events; and (B) only fatal vascular events. Area of each square is proportional to the inverse variance of the estimate.

**Figure 4:**
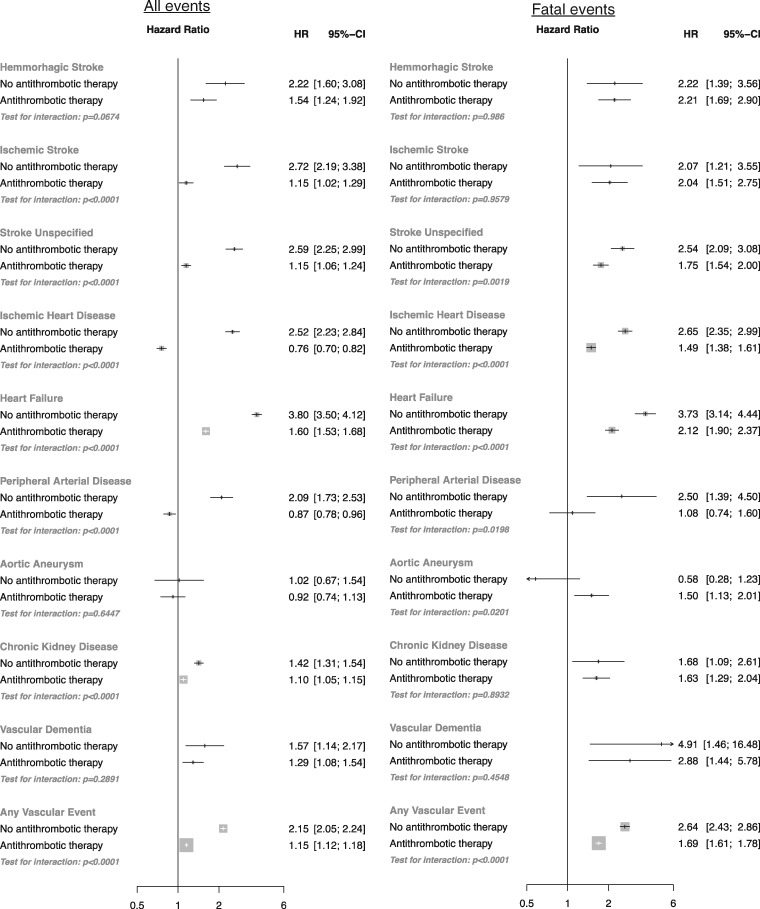
Adjusted hazard ratios of baseline atrial fibrillation for nine different vascular events. Adjustments were for age, BMI, smoking status, sex, socioeconomic status, baseline diabetes, baseline antihypertensive use, baseline lipid-lowering drug (statin) use, baseline anticoagulant usage, baseline antiplatelet usage and the interaction between baseline atrial fibrillation and baseline anticoagulant or antiplatelet use (plotted). Restricted to: (A) fatal and non-fatal vascular events; and (B) only fatal vascular events. Area of each square is proportional to the inverse variance of the estimate.

Overall, baseline AF was associated with a 31% higher risk of any vascular event (HR 1.31, CI 1.28, 1.34) and a 89% higher risk of a fatal vascular event (HR 1.89, CI 1.81, 1.96, Figure 3). However, an interaction was observed by baseline usage of antithrombotic therapy (Figure 4). Whereas AF with baseline antithrombotic therapy usage was associated with a 15% higher risk of any vascular event (HR 1.15, CI 1.12, 1.18) and a 69% higher risk of a fatal vascular event (HR 1.69, CI 1.61, 1.78), AF without antithrombotic therapy was associated with two times the risk of any vascular event (HR 2.15, CI 2.05, 2.24, *P* interaction < 0.0001) and two and a half times the risk of a fatal vascular event (HR 2.64, CI 2.43, 2.86). AF without antithrombotic therapy was associated with an increased risk of ischaemic stroke (HR 2.72, CI 2.19, 3.38), stroke unspecified (HR 2.59, CI 2.25, 2.99), ischaemic heart disease (HR 2.52, CI 2.23, 2.84), heart failure (HR 3.80, CI 3.50, 4.12), peripheral arterial disease (2.09 CI, 1.73, 2.53) and chronic kidney disease (HR 1.42, CI 1.31, 1.54) relative to AF with baseline antithrombotic therapy use (all *P* interaction < 0.001). Risks of haemorrhagic stroke and vascular dementia were associated with AF but did not differ by baseline antithrombotic therapy usage. An increased risk of fatal aortic aneurysm was observed among individuals with AF and antithrombotic usage (but not with atrial fibrillation without antithrombotic usage).

**Figure 5:**
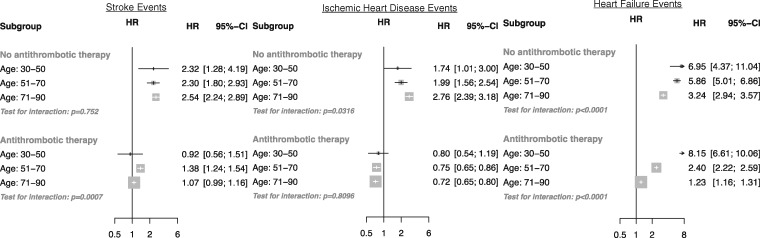
Adjusted hazard ratios of baseline atrial fibrillation for stroke, ischaemic heart disease and heart failure. Adjustments were for age category, interaction between continuous age and age category, BMI, smoking status, sex, baseline diabetes, baseline antihypertensive use, baseline lipid- lowering drug (statin) use and the interaction between baseline atrial fibrillation, age category and baseline anticoagulant/antiplatelet usage (plotted). Area of each square is proportional to the inverse variance of the estimate.

Overall, the risk of any stroke (ischaemic, haemorrhagic or unspecified) was associated with AF without antithrombotic usage (HR 2.55, CI 2.28, 2.86) and AF with antithrombotic usage (HR 1.24, CI 1.22, 1.27). Although the HR of AF for stroke did not increase with age, the HR for AF-associated ischaemic heart disease in patients who were not taking antithrombotic therapy was directly associated with age (Figure 5). In contrast, the HR for heart failure associated with AF declined with increasing age.

Estimates for each outcome (Supplementary Figures 9-16, available as Supplementary data at *IJE* online) were broadly similar within each of our sensitivity analyses.

## Discussion

In this analysis of 4.3 million individuals and 128 468 incident AF events, SBP and DBP were positively related to risk of AF. At age 30-40 years, 20 mmHg higher SBP was associated with a near doubling in risk of AF. Although proportional associations declined with age, the absolute difference in risk of AF associated with a 20-mmHg higher SBP increased until approximately age 51-60, due to the much greater baseline risk of AF in middle and older age.

Previous analyses of the relationship between BP and AF have generally concluded that elevated BP is a risk factor for AF (Supplementary Table 1). In an analysis of 5201 individuals in the Cardiovascular Health Study, 20-mmHg higher SBP was associated with a 23% increased risk of atrial fibrillation (HR 1.23, CI 1.10, 1.39), highly similar to the 21% increased risk per 20 mmHg higher SBP observed in this study. Analyses of the Framingham^30^ and the Manitoba Follow-up Study^31^ similarly concluded that hypertension is a risk factor for atrial fibrillation. The findings from the present much larger study confirm earlier reports. In addition, they extend previous findings by demonstrating that the observed association between BP and AF varies by age, which previous smaller studies have failed to detect.^32^^,^^33^ The interaction between BP and age is similar to what has been reported for other vascular outcomes.^16^^,^^34^ The present study shows that, for example, the absolute risk of AF attributable to high blood pressure is on average higher in a middle-aged person than in a young person despite stronger relative risk associations in the younger age group. On the other hand, in elderly people the very high absolute risk of developing AF is less strongly affected by differences in baseline blood pressure. The understanding of these differential effects of blood pressure on risk of AF will help to inform communication of risks and adaptation of treatment strategies, as an important step towards stratified medicine.

The second focus of this study was the analysis of risk of AF for a range of vascular events. In our analysis of 421 084 incident vascular events, AF was observed to be a risk factor for cardiovascular disease. However, an interaction was observed by baseline usage of antithrombotic therapy (antiplatelet or anticoagulant therapies). AF without usage of antithrombotic therapy was associated with a two to three times proportional risk of haemorrhagic stroke, ischaemic stroke and stroke unspecified, a two and a half times proportional risk of ischaemic heart disease, a near four times proportional risk of heart failure, and increased risk of peripheral arterial disease, chronic kidney disease and vascular dementia. However, no association was observed between atrial fibrillation and risk of aortic aneurysm. Although an increased risk of fatal aortic aneurysm was observed among individuals with AF and antithrombotic usage, this may have been due to chance, as no increased risk was observed for fatal and non-fatal aortic aneurysm.

Previous analyses of the relationship between AF and vascular risk have largely focused on stroke (Supplementary Table 1). In the Framingham cohort, AF was associated with 2.6 times the risk of stroke at age 60-69, and 4.5 times the risk at age 80-89.^4^ However, this analysis largely preceded the widespread use of antithrombotic therapies as well as antihypertensive and lipid-lowering therapies. Indeed, in an analysis of a contemporary registry with widespread use of antithrombotic, blood pressure-lowering and lipid-lowering agents, AF was associated with an unadjusted 1.6-fold risk of non-fatal stroke.^35^ In an analysis from the Manitoba Follow-Up Study, AF was associated with a 2-fold risk of stroke (HR 2.07).^31^ Our estimate of a 2.5 times risk of stroke associated with AF without baseline antithrombotic usage lies between these estimates and the older Framingham estimates.

However, in addition to observing an expected association between AF and risk of stroke, we also observed AF to be associated with an increased risk of a variety of vascular events including ischaemic heart disease, chronic kidney disease, peripheral arterial disease and vascular dementia. Previous reports on the relationship between AF and cardiovascular mortality have indicated that AF is associated with an increased risk of cardiovascular death, with stroke deaths composing a minority of excess deaths.^7^ Our results substantiate these previous observations and findings from more recent cohort studies indicating that AF is associated with an increased risk of myocardial infarction^36^^,^^37^ and other non-stroke vascular causes of death (Supplementary Table 1). A recent analysis suggested that AF is associated with non-ST-segment elevated myocardial infarction but not ST-segment-elevated myocardial infarction,^38^ an association that may be mediated by haemostatic factors.^39^ Recent analyses have also suggested that AF is associated with incident chronic kidney disease^40^ and end-stage renal disease.^41^

This report has several strengths. It is based on a very large sample, encompassing more than 4 million individuals, 100 0000 incident AF events and 400 000 vascular events, allowing for more detailed analyses than previously possible. It is also contemporary, with many individuals using anticoagulant, antiplatelet, antihypertensive and lipid-lowering therapies, unlike in many previous reports.^42^

A potential limitation is that we used routinely collected electronic health records for our analysis and our events were not adjudicated. Although this approach has been previously validated for epidemiological research^9^ and used to examine the associations between (among others) blood pressure^10^ and type 2 diabetes^43^ and vascular risk, the risk of misclassification of events in such an approach is likely to be higher than in a traditional cohort study. However, we undertook a number of sensitivity analyses and found no changes in the inferences drawn. Indeed, in the large-scale Prospective Studies Collaboration^16^ and Emerging Risk Factors Collaboration,^44^ ascertainment of fatal events, including stroke subtypes as ischaemic, haemorrhagic and unspecified, was based on death certificates, as it was for our linked cause-specific mortality data.

A further limitation is the potential for confounding due to the observational nature of this analysis. Indeed, the observed increased risk of a variety of vascular events associated with AF may, in part, be due to undiagnosed vascular disease (which we therefore could not exclude) or other undiagnosed comorbidities among patients with AF which we were unable to adjust for. Differences in ethnicity may also have confounded reported estimates. Similarly, the differences in risk of vascular events by baseline antithrombotic usage may be due, in part, to confounding by indication. Individuals prescribed antithrombotic therapy may be more likely to receive better clinical care or be at lower risk for a vascular event.

An additional limitation is that the CPRD population has previously been shown to be predominantly Caucasian, and the associations between BP, AF and vascular risk may differ by ethnicity.^12^

Finally, measurement error in both BP and AF may differ by age or other patient characteristics, which may bias observed associations.

Our findings have several possible clinical and research implications. The age-specific associations provided in this analysis between BP, AF and vascular risk may allow clinicians to better personalize management plans. They further help to inform the design of interventional studies for management of vascular risk in patients with AF. The findings raise the hypothesis that treatments for patients with established AF could have beneficial effects on reducing the risk of a range of vascular events, not only stroke. In particular, lower rates of ischaemic heart disease and heart failure were observed with baseline antithrombotic usage, an association that, if causal, would represent a benefit largely unattributed to the provision of antithrombotic therapy in atrial fibrillation. Indeed, a recent meta-analysis of randomized trials of anticoagulant use demonstrated that anticoagulant use reduces the risk of myocardial infarction,^45^ highlighting the importance of anticoagulant use for prevention of non-stroke cardiovascular disease. An individual patient data meta-analysis of placebo- controlled warfarin trials in atrial fibrillation may allow this hypothesis to be tested for other vascular outcomes.

### Conclusions

Although 20 mmHg higher systolic blood pressure was on average associated with a 21% higher risk of AF, the association varied substantially by age. AF was further found to be a risk factor for a range of fatal and non-fatal vascular events, suggesting that existing and novel treatments forAF may have greater beneficial effects than currently assumed. Further research should determine whether the described associations are causal.

## Supplementary Data

Supplementary data are available at *IJE* online.

## Funding

K.R. is supported by the NIHR Oxford Biomedical Research Centre and NIHR Career Development Fellowship. C.E. is supported by the Rhodes Trust. M.W. is supported by a Principal Research Fellowship from the Australian Health and Medical Research Council. The work of the George Institute is supported by the Oxford Martin School. S.A. is an academic clinical lecturer in cardiology and is funded by the National Institute of Health Research. This work was funded by the UK National Institute for Health Research. The funder had no role in study design, data collection and analysis, decision to publish or preparation of the manuscript.

**Conflict of interest:** M.W. reports consulting fees from Amgen, Novartis and Roche, and grant support from Roche. All other authors report no competing interests.

Key messages

- This paper examines the association between blood pressure and risk of incident atrial fibrillation and the association between atrial fibrillation and risk of incident cardiovascular disease.
- A 20-mmHg higher usual systolic blood pressure was associated with a 21% higher risk of AF. However, the strength of the association declined with increasing age.
- AF without antithrombotic use at baseline was associated with a greater risk of any vascular event than AF with antithrombotic usage (*P* interaction < 0.0001).
- AF without baseline antithrombotic usage was associated with an increased risk of ischaemic heart disease (HR 2.52, CI 2.23, 2.84), heart failure (HR 3.80, CI 3.50, 4.12), ischaemic stroke (HR 2.72, CI 2.19, 3.38), unspecified stroke (HR 2.59, CI 2.25, 2.99), haemorrhagic stroke, chronic kidney disease, peripheral arterial disease and vascular dementia, but not aortic aneurysm.

## Supplementary Material

Supplementary Data
