# Supplementary material for: Usual blood pressure, atrial fibrillation and vascular risk: evidence from 4.3 million adults
Source: Int J Epidemiol. 2016 Apr 28;46(1):162–72. doi: 10.1093/ije/dyw053 (PMC5407172; doi:10.1093/ije/dyw053)
Supplement: Supplementary Data [file dyw053_supp.docx]

# Supplementary Appendix

Supp. Table 1. Previous major studies on the relationship between blood pressure and incident atrial fibrillation and the relationship between atrial fibrillation and vascular risk.

| Study | Size | Design | Results | Interpretation |
| --- | --- | --- | --- | --- |
| **Blood pressure as a risk factor for atrial fibrillation** | | | |  |
| Emdin et al. (Current study) | 4.3 million individuals/128468 atrial fibrillation events | Cohort study using linked electronic health records | 20 mm Hg higher SBP was associated with HR 1.22 (CI 1.21, 1.24) risk of atrial fibrillation; declined from HR 1.93 (CI 1.75, 2.13) at age 30-40 to HR | Higher blood pressure associated with higher risk of atrial fibrillation; strength of association declines with age |
| Alonso et al.^1^ | 18 556 individuals/1771 | Pooled analysis of three cohorts | 20 mm Hg higher SBP associated with a HR 1.20 (CI 1.13, 1.29) risk of AT | Higher blood pressure associated with higher risk of AF |
| Conen et al. ^2^ | 34 221 women/644 atrial fibrillation events | Cohort study (Women’s Health Study) | 10 mm Hg higher SBP associated with HR 1.16 (CI 1.09, 1.23) for AF. Elevated BP in non-hypertensive range associated with increased risk AF. | Higher blood pressure associated with increased risk of AF, even in non-hypertensive range. |
| Kannel et al.^3^ | 5191 individuals/98 atrial fibrillation events | Cohort study (Framingham) | Hypertension (SBP > 160/95 mm Hg or antihypertensives) associated with non-significant risk ratio of 1.6 in men and 1.8 in women | Hypertension is not associated with risk of atrial fibrillation |
| Krahn et al.^4^ | 3983 individuals/299 atrial fibrillation events | Cohort study (Manitoba Follow-up Study) | Hypertension associated with HR 1.42 CI 1.10, 1.84 risk of atrial fibrillation after multivariable adjustment | Hypertension is associated with increased risk of atrial fibrillation |
| Nyrnes et al.^5^ | 22 815 individuals/822 atrial fibrillation events | Cohort study (The Tromsø Study). Used linked electronic health records | Hypertension was defined as systolic blood pressure (SBP) >140 mmHg *or* diastolic blood pressure (DBP) >  90 mmHg *or* use of antihypertensive treatment associated with significant risk of 1.98 in women and 1.4 in men | Elevated systolic blood pressure associated with increased risk of atrial fibrillation |
| Psaty et al.^6^ | 5201 individuals/304 atrial fibrillation events | Cohort study (Cardiovascular Health Study) | 10 mm Hg higher SBP was associated with HR 1.11 (CI 1.05, 1.18) for risk of atrial fibrillation | Higher SBP associated with higher risk of atrial fibrillation |
| Roetker et al.^7^ | 6630 individuals/307 atrial fibrillation events | Cohort study (Multi-Ethnic Study of Atherosclerosis) | 1 SD increase in SBP (21.5 mm Hg) associated with HR 1.16 (CI 1.03, 1.31) | Higher SBP associated with higher risk of atrial fibrillation |
| Smith et al.^8^ | 30447 individuals/1430 events | Cohort study (Malmo Diet and Cancer Study) | Hypertension (BP > 140/90 mm Hg) associated with a HR 1.78 (CI 1.48, 2.14) for risk of AF | Hypertension associated with higher risk of AF |
| Wilhelmsen et al.^9^ | 7495/754 atrial fibrillation events | Cohort study, hospitalization for atrial fibrillation as primary outcome | Relative to SBP < 145 mm Hg, SBP 145-175 and SBP > 175 mm Hg associated with OR 1.30 (CI 1.11, 1.54) and OR 1.37 (CI 1.09, 1.74) respectively | Elevated systolic blood pressure associated with increased risk of atrial fibrillation |
| **Atrial fibrillation as a risk factor for vascular events** | | | |  |
| Emdin et al. (Current study) | 4.3 million individuals/421 084 vascular events |  | Baseline atrial fibrillation associated with increased risk of ischemic stroke, hemmorhagic stroke, stroke unspecified, ischemic heart disease, heart failure, peripheral arterial disease, chronic kidney disease vascular dementia but not aortic aneurysm. Significantly greater risk of ischemic stroke, strok unspecified, ischemic heart disease, heart failure, peripheral aterial disease and chronic kidney disease observed among individuals who did not take antithrombotic therapy at baseline. | Atrial fibrillation is a risk factor for a range of vascular events; |
| Bansal et al. 2013 ^10^ | 206 229 individuals/6850 ESRD events | Cohort study using administrative data (Kaiser Permanente) | Among patients with already developed CKD, atrial fibrillation associated with HR 1.67 (CI 1.46, 1.91) for progression of ESRD. | Atrial fibrillation a significant risk factor for progression of CKD to ESRD. |
| Watanabe et al. 2013 ^11^ | 235 818 individuals/7791 CKD events | Cohort study (Niigata Preventive Medicine Study) | Among patients without CKD, AF associated with HR 1.80 (CI 1.54, 2.10) for development of CKD. | Atrial fibrillation a significant risk for incident CKD. |
| Conen et al.^12^ | 34 772 participants/309 cardiovascular deaths | Cohort study (Women’s Health Study) | After exclusion of early deaths, incident atrial fibrillation associated with increased risk of stroke and heart failure, but not myocardial infarction. | Atrial fibrillation is a risk factor for stroke, heart failure, but not myocardial infarction. |
| Chen et al. ^13^ | 15 439 participants/1375 coronary heart disease fatal events | Cohort study (Atherosclerosis Risk in Communities Study and Cardiovascular Health Study) | Incident atrial fibrillation associated with an increased risk of sudden cardiac death and non-sudden cardiac death. | Atrial fibrillation a risk factor for coronary heart disease death. |
| Soliman et al.^14^ | 23 928 participants/648 incident myocardial infarction events | Cohort study (Reasons for Geographic and Racial Differences in Stroke) | Baseline atrial fibrillation associated with HR 1.96 CI for incident myocardial infarction after socio-demographic adjustment | Atrial fibrillation is a risk factor for myocardial infarction. |
| Wolf et al. 1978^15^ | 5184/345 strokes | Cohort study (Framingham) | Atrial fibrillation associated with 5.6 fold increase in rate of stroke (p<0.01). | Atrial fibrillation is a significant risk factor for stroke. |
| Wolf et al. 1991^16^ | 5070 individuals/527 strokes | Cohort study (Framingham) | Atrial fibrillation associated with near five fold excess risk of stroke; relative risk increases from 2.6 (p<0.001) at age 60-69 to 4.5 (p<0.001) at age 80-89 | Atrial fibrillation is a significant risk factor for stroke, association increases with age. |

Supp. Table 2. Primary and secondary endpoints used in the analysis.^17^

|  | Definition |  | ICD-10 Codes | ICD-9 Codes | Prior Validation Studies of Disease in CPRD |
| --- | --- | --- | --- | --- | --- |
| *Primary outcome* |  |  |  |  |  |
| Atrial fibrillation | Incident diagnosis of, hospitalization or death due to atrial fibrillation or atrial flutter, no restrictions on type. |  | I48 (fatal and non-fatal) | 427.3 (fatal and non-fatal), | Similar association with risk factors as from traditional cohort studies.^18^ |
| *Secondary outcomes* |  |  |  |  |  |
| Aortic aneurysm | Incident diagnosis of, hospitalization or death due to aortic aneurysm. |  | I71 (fatal and non-fatal) | 441 (fatal and non-fatal), | NA |
| Chronic kidney disease | Incident diagnosis of, hospitalization or death due to stage 3-5 chronic kidney disease. |  | N18, I12, I13, E08.22, E09.22, E10.22, E11.22, E13.22 (fatal and non-fatal) | 403, 404 (fatal and non-fatal) | Similar prevalence in CPRD to national cross-sectional surveys.^19^ |
| Ischemic heart disease | Incident diagnosis of, hospitalization non-fatal myocardial infarction or death due to ischemic heart disease. |  | I20-I25(fatal), I21-I23(non-fatal) | 410-414(fatal), 410-412 (fatal and non-fatal) | PPV: 92.6%^20^ |
| Heart failure | Incident diagnosis of, hospitalization or death due to heart failure. |  | I11.0, I13.0, I13.2, I50 (fatal and non-fatal) | 428, 402.01, 402.11, 402.91, 404.01, 404.11, 404.91, 404.03, 404.13  404.93 (fatal and non-fatal) | PPV: 81.8%^21^ |
| Peripheral arterial disease | Incident diagnosis of peripheral arterial disease, including intermittent claudication, hospitalization or death due to peripheral arterial disease. |  | I73.1, I73.8, I73.9, I74.3, I74.4, I74.5 (fatal and non-fatal) | 443.1, 443.8, 443.9, 444.22, 444.81 (fatal and non-fatal) | Similar association with cardiovascular risk factors as traditional cohort studies.^22,23^ Median PPV of 85% for GPRD coding of circulatory disorders.^24^ |
| Stroke | Incident diagnosis of, hospitalization or death due to stroke. |  | I60, I61, I63, I64 (fatal and non-fatal), I67.2, I67.9 (fatal only) | 430, 431, 434, 437 (fatal and non-fatal) | PPV: 92.7%^20^ |

Supp. Table 3. Incident atrial fibrillation diagnoses (per 1000 patient years) by age and sex.

| Age | Male | Female |
| --- | --- | --- |
| 30-40 | 0.6 | 0.2 |
| 41-50 | 1.6 | 0.8 |
| 51-60 | 4.4 | 2.5 |
| 61-70 | 10.1 | 7.2 |
| 71-80 | 19.0 | 15.1 |
| 81-90 | 28.4 | 23.8 |

Supp. Table 4. Characteristics of participants included in the analysis of the association between baseline atrial fibrillation and vascular risk.

|  | **No atrial fibrillation** | **Atrial fibrillation** | **Overall** |
| --- | --- | --- | --- |
| N | 4269194 | 32 155 | 4 301 349 |
| Age at Baseline | 46 (IQI 36, 59) | 72 (IQI 61, 80) | 47 (IQI 36, 60) |
| Women | 2 374 239 (55.6%) | 14 751(45.9%) | 2 388 990 (55.5%) |
| BMI | 25.8 (IQI 23.0, 29.3) | 26.1 (IQI 23.1, 29.6) | 25.8 (IQI 23.0, 29.3) |
| Smoking Status |  |  |  |
| Current Smoker | 970 374 (28.0%) | 7006 (28.9%) | 973 802 (27.9%) |
| Never Smoker | 616 254 (17.8%) | 3428 (14.1%) | 623 260 (17.8%) |
| Ex Smoker | 1883584 (54.3%) | 13829 (57.0%) | 1 897 413 (54.3%) |
| Cholesterol |  |  |  |
| Total | 5.5 (IQI 4.7, 6.2) | 5.1 (IQI 4.4, 5.9) | 5.5 (IQI 4.7, 6.2) |
| HDL | 1.4 (IQI 1.1, 1.6) | 1.3 (IQI 1.1, 1.6) | 1.4 (IQI 1.1, 1.6) |
| Most deprived quintile | 807 983 (18.9%) | 4969 (15.5%) | 812 952 (18.9%) |
| Antihypertensive at baseline | 431045 (10.1%) | 12 705 (39.5%) | 443 750 (10.3%) |
| Antihypertensive during follow up | 1235995 (29.0%) | 20450 (63.6%) | 1256445 (29.2%) |
|  |  |  |  |
| Lipid lowering at baseline | 82031 (1.9%) | 2300 (7.2%) | 84 331 (2.0%) |
| Lipid lowering during follow up | 665 879 (15.6%) | 8731 (27.2%) | 674 610 (15.7%) |
| Anticoagulant at baseline | 150 081 (3.5%) | 12 972 (40.3%) | 163 053 (3.8%) |
| Antiplatelet at baseline | 610 427 (14.3%) | 15 398 (47.9%) | 625 825 (14.5%) |
| Diabetes at baseline | 137 468 (3.2%) | 2758 (8.6%) | 140 226 (3.3%) |

IQI refers to interquartile interval. Proportion of patients with missing covariates: BMI (30.6%), smoking (18.8%), total cholesterol (72.8%), HDL cholesterol (80.0%)

- 24 280 were above the age of 90 at baseline

4 301 349 individuals remaining

- 32 155 had atrial fibrillation at baseline
- 128 468 developed new-onset atrial fibrillation during follow up
- 421 084 had a non-atrial fibrillation vascular event during follow up

15 236 931 individuals in CPRD (January 2014)

4 737 123 individuals remaining

10 499 808 were not:

- over age of 30
- had a BP measurement
- had at least one year of follow up in CPRD prior to study entry
- 411 494 had pre-existing existing cardiovascular disease

4 325 629 individuals remaining

Supp. Figure 1. Flowchart of cohort identification.

Supp. Figure 2. Adjusted hazard ratios of atrial fibrillation per 10 mm Hg higher usual DBP by patient subgroup. Adjustments were for age, BMI, smoking status, sex and baseline diabetes. Further adjustments were for age category and the interaction between systolic BP and age category (plotted, for subgroup Age), the interaction between sex and systolic BP (plotted, for subgroup Sex), and BMI category and the interaction between systolic BP and BMI category (plotted, for subgroup BMI). Area of each square is proportional to the inverse variance of the estimate.

Supp. Figure 3. Adjusted hazard ratios of atrial fibrillation per 20 mm Hg higher usual SBP by patient subgroup. Adjustments were for age, BMI, smoking status, sex, baseline diabetes, total cholesterol and HDL cholesterol. Further adjustments were for age category and the interaction between systolic BP and age category (plotted, for subgroup Age), the interaction between sex and systolic BP (plotted, for subgroup Sex), and BMI category and the interaction between systolic BP and BMI category (plotted, for subgroup BMI). Area of each square is proportional to the inverse variance of the estimate.

Supp. Figure 4. Adjusted hazard ratios of atrial fibrillation per 20 mm Hg higher usual SBP by patient subgroup. Adjustments were for age, BMI, smoking status, sex and baseline diabetes, total cholesterol, HDL cholesterol and period of blood pressure measurement. Further adjustments were for the interaction between systolic BP and age category (plotted, for subgroup Age), the interaction between sex and systolic BP (plotted, for subgroup Sex), and BMI category and the interaction between systolic BP and BMI category (plotted, for subgroup BMI). Area of each square is proportional to the inverse variance of the estimate.

Supp. Figure 5. Adjusted hazard ratios of atrial fibrillation per 20 mm Hg higher usual SBP by patient subgroup. Adjustments were for age, BMI, smoking status, sex and baseline diabetes. Further adjustments were for age category and the interaction between systolic BP and age category (plotted, for subgroup Age), the interaction between sex and systolic BP (plotted, for subgroup Sex), and BMI category and the interaction between systolic BP and BMI category (plotted, for subgroup BMI). First two years of follow up are excluded. Area of each square is proportional to the inverse variance of the estimate.

Supp. Figure 6. Adjusted hazard ratios of atrial fibrillation per 20 mm Hg higher usual SBP by patient subgroup. Adjustments were for age, BMI, smoking status, sex and baseline diabetes. Further adjustments were for age category and the interaction between systolic BP and age category (plotted, for subgroup Age), the interaction between sex and systolic BP (plotted, for subgroup Sex), and BMI category and the interaction between systolic BP and BMI category (plotted, for subgroup BMI). First four years of follow up are excluded. Area of each square is proportional to the inverse variance of the estimate.

Supp. Figure 7. Adjusted hazard ratios of atrial fibrillation per 20 mm Hg higher usual SBP by patient subgroup. Adjustments were for age, BMI, smoking status, sex, baseline diabetes, baseline antihypertensive use, baseline lipid lowering drug (statin) use. Further adjustments were for age category and the interaction between systolic BP and age category (plotted, for subgroup Age), the interaction between sex and systolic BP (plotted, for subgroup Sex), and BMI category and the interaction between systolic BP and BMI category (plotted, for subgroup BMI). Individuals prescribed antihypertensive medication at baseline are excluded. Area of each square is proportional to the inverse variance of the estimate.

Supp. Figure 8. Adjusted hazard ratios of atrial fibrillation per 20 mm Hg higher usual SBP by patient subgroup. Adjustments were for age, BMI, smoking status, sex, baseline diabetes, baseline antihypertensive use, baseline lipid lowering drug (statin) use. Further adjustments were for age category and the interaction between systolic BP and age category (plotted, for subgroup Age), the interaction between sex and systolic BP (plotted, for subgroup Sex), and BMI category and the interaction between systolic BP and BMI category (plotted, for subgroup BMI). Atrial fibrillation was restricted to diagnosis of “Atrial fibrillation” in primary care. Area of each square is proportional to the inverse variance of the estimate.

Supp. Figure 9. Association of baseline atrial fibrillation with the risk of hemorrhagic stroke. Primary adjustments (1) were for age, BMI, smoking status, sex, baseline diabetes, baseline antihypertensive use, baseline lipid lowering drug (statin) use, baseline anticoagulant usage and baseline antiplatelet usage and interaction between atrial fibrillation and anticoagulant or antiplatelet usage (plotted). Sensitivity analyses were: (2) further adjustment for total cholesterol and HDL cholesterol, (3) further adjustment for total cholesterol, HDL cholesterol and period of blood pressure measurement, (4) exclusion of the first two years of follow up, (5) exclusion of the first four years of follow up, (6) exclusion of individuals who presented with atrial fibrillation three years or more before the baseline blood pressure measurement and have not been diagnosed with atrial fibrillation in the three years prior to the baseline blood pressure measurement, (7) exclusion individuals who developed atrial fibrillation during follow up, (8) restriction of atrial fibrillation to diagnosis of “Atrial fibrillation” in primary care, (9) exclusion of participants on antiplatelet therapy and (10) exclusion of participants on anticoagulant therapy. Area of each square is proportional to the inverse variance of the estimate.

Supp. Figure 10. Association of baseline atrial fibrillation with the risk of ischemic stroke. Primary adjustments (1) were for age, BMI, smoking status, sex, baseline diabetes, baseline antihypertensive use, baseline lipid lowering drug (statin) use, baseline anticoagulant usage and baseline antiplatelet usage and interaction between atrial fibrillation and anticoagulant or antiplatelet usage (plotted). Sensitivity analyses were: (2) further adjustment for total cholesterol and HDL cholesterol, (3) further adjustment for total cholesterol, HDL cholesterol and period of blood pressure measurement, (4) exclusion of the first two years of follow up, (5) exclusion of the first four years of follow up, (6) exclusion of individuals who presented with atrial fibrillation three years or more before the baseline blood pressure measurement and have not been diagnosed with atrial fibrillation in the three years prior to the baseline blood pressure measurement, (7) exclusion individuals who developed atrial fibrillation during follow up, (8) restriction of atrial fibrillation to diagnosis of “Atrial fibrillation” in primary care, (9) exclusion of participants on antiplatelet therapy and (10) exclusion of participants on anticoagulant therapy. Area of each square is proportional to the inverse variance of the estimate.

Supp. Figure 11. Association of baseline atrial fibrillation with the risk of stroke unspecified. Primary adjustments (1) were for age, BMI, smoking status, sex, baseline diabetes, baseline antihypertensive use, baseline lipid lowering drug (statin) use, baseline anticoagulant usage and baseline antiplatelet usage and interaction between atrial fibrillation and anticoagulant or antiplatelet usage (plotted). Sensitivity analyses were: (2) further adjustment for total cholesterol and HDL cholesterol, (3) further adjustment for total cholesterol, HDL cholesterol and period of blood pressure measurement, (4) exclusion of the first two years of follow up, (5) exclusion of the first four years of follow up, (6) exclusion of individuals who presented with atrial fibrillation three years or more before the baseline blood pressure measurement and have not been diagnosed with atrial fibrillation in the three years prior to the baseline blood pressure measurement, (7) exclusion individuals who developed atrial fibrillation during follow up, (8) restriction of atrial fibrillation to diagnosis of “Atrial fibrillation” in primary care, (9) exclusion of participants on antiplatelet therapy and (10) exclusion of participants on anticoagulant therapy. Area of each square is proportional to the inverse variance of the estimate.

Supp. Figure 12. Association of baseline atrial fibrillation with the risk of ischemic heart disease. Primary adjustments (1) were for age, BMI, smoking status, sex, baseline diabetes, baseline antihypertensive use, baseline lipid lowering drug (statin) use, baseline anticoagulant usage and baseline antiplatelet usage and interaction between atrial fibrillation and anticoagulant or antiplatelet usage (plotted). Sensitivity analyses were: (2) further adjustment for total cholesterol and HDL cholesterol, (3) further adjustment for total cholesterol, HDL cholesterol and period of blood pressure measurement, (4) exclusion of the first two years of follow up, (5) exclusion of the first four years of follow up, (6) exclusion of individuals who presented with atrial fibrillation three years or more before the baseline blood pressure measurement and have not been diagnosed with atrial fibrillation in the three years prior to the baseline blood pressure measurement, (7) exclusion individuals who developed atrial fibrillation during follow up, (8) restriction of atrial fibrillation to diagnosis of “Atrial fibrillation” in primary care, (9) exclusion of participants on antiplatelet therapy and (10) exclusion of participants on anticoagulant therapy. Area of each square is proportional to the inverse variance of the estimate.

Supp. Figure 13. Association of baseline atrial fibrillation with the risk of heart failure. Primary adjustments (1) were for age, BMI, smoking status, sex, baseline diabetes, baseline antihypertensive use, baseline lipid lowering drug (statin) use, baseline anticoagulant usage and baseline antiplatelet usage and interaction between atrial fibrillation and anticoagulant or antiplatelet usage (plotted). Sensitivity analyses were: (2) further adjustment for total cholesterol and HDL cholesterol, (3) further adjustment for total cholesterol, HDL cholesterol and period of blood pressure measurement, (4) exclusion of the first two years of follow up, (5) exclusion of the first four years of follow up, (6) exclusion of individuals who presented with atrial fibrillation three years or more before the baseline blood pressure measurement and have not been diagnosed with atrial fibrillation in the three years prior to the baseline blood pressure measurement, (7) exclusion individuals who developed atrial fibrillation during follow up, (8) restriction of atrial fibrillation to diagnosis of “Atrial fibrillation” in primary care, (9) exclusion of participants on antiplatelet therapy and (10) exclusion of participants on anticoagulant therapy. Area of each square is proportional to the inverse variance of the estimate.

Supp. Figure 14. Association of baseline atrial fibrillation with the risk of peripheral arterial disease. Primary adjustments (1) were for age, BMI, smoking status, sex, baseline diabetes, baseline antihypertensive use, baseline lipid lowering drug (statin) use, baseline anticoagulant usage and baseline antiplatelet usage and interaction between atrial fibrillation and anticoagulant or antiplatelet usage (plotted). Sensitivity analyses were: (2) further adjustment for total cholesterol and HDL cholesterol, (3) further adjustment for total cholesterol, HDL cholesterol and period of blood pressure measurement, (4) exclusion of the first two years of follow up, (5) exclusion of the first four years of follow up, (6) exclusion of individuals who presented with atrial fibrillation three years or more before the baseline blood pressure measurement and have not been diagnosed with atrial fibrillation in the three years prior to the baseline blood pressure measurement, (7) exclusion individuals who developed atrial fibrillation during follow up, (8) restriction of atrial fibrillation to diagnosis of “Atrial fibrillation” in primary care, (9) exclusion of participants on antiplatelet therapy and (10) exclusion of participants on anticoagulant therapy. Area of each square is proportional to the inverse variance of the estimate.

Supp. Figure 15. Association of baseline atrial fibrillation with the risk of aortic aneurysm. Primary adjustments (1) were for age, BMI, smoking status, sex, baseline diabetes, baseline antihypertensive use, baseline lipid lowering drug (statin) use, baseline anticoagulant usage and baseline antiplatelet usage and interaction between atrial fibrillation and anticoagulant or antiplatelet usage (plotted). Sensitivity analyses were: (2) further adjustment for total cholesterol and HDL cholesterol, (3) further adjustment for total cholesterol, HDL cholesterol and period of blood pressure measurement, (4) exclusion of the first two years of follow up, (5) exclusion of the first four years of follow up, (6) exclusion of individuals who presented with atrial fibrillation three years or more before the baseline blood pressure measurement and have not been diagnosed with atrial fibrillation in the three years prior to the baseline blood pressure measurement, (7) exclusion individuals who developed atrial fibrillation during follow up, (8) restriction of atrial fibrillation to diagnosis of “Atrial fibrillation” in primary care, (9) exclusion of participants on antiplatelet therapy and (10) exclusion of participants on anticoagulant therapy. Area of each square is proportional to the inverse variance of the estimate.

Supp. Figure 16. Association of baseline atrial fibrillation with the risk of chronic kidney disease. Primary adjustments (1) were for age, BMI, smoking status, sex, baseline diabetes, baseline antihypertensive use, baseline lipid lowering drug (statin) use, baseline anticoagulant usage and baseline antiplatelet usage and interaction between atrial fibrillation and anticoagulant or antiplatelet usage (plotted). Sensitivity analyses were: (2) further adjustment for total cholesterol and HDL cholesterol, (3) further adjustment for total cholesterol, HDL cholesterol and period of blood pressure measurement, (4) exclusion of the first two years of follow up, (5) exclusion of the first four years of follow up, (6) exclusion of individuals who presented with atrial fibrillation three years or more before the baseline blood pressure measurement and have not been diagnosed with atrial fibrillation in the three years prior to the baseline blood pressure measurement, (7) exclusion individuals who developed atrial fibrillation during follow up, (8) restriction of atrial fibrillation to diagnosis of “Atrial fibrillation” in primary care, (9) exclusion of participants on antiplatelet therapy and (10) exclusion of participants on anticoagulant therapy. Area of each square is proportional to the inverse variance of the estimate.

Supp. Figure 17. Association of baseline atrial fibrillation with the risk of vascular dementia. Primary adjustments (1) were for age, BMI, smoking status, sex, baseline diabetes, baseline antihypertensive use, baseline lipid lowering drug (statin) use, baseline anticoagulant usage and baseline antiplatelet usage and interaction between atrial fibrillation and anticoagulant or antiplatelet usage (plotted). Sensitivity analyses were: (2) further adjustment for total cholesterol and HDL cholesterol, (3) further adjustment for total cholesterol, HDL cholesterol and period of blood pressure measurement, (4) exclusion of the first two years of follow up, (5) exclusion of the first four years of follow up, (6) exclusion of individuals who presented with atrial fibrillation three years or more before the baseline blood pressure measurement and have not been diagnosed with atrial fibrillation in the three years prior to the baseline blood pressure measurement, (7) exclusion individuals who developed atrial fibrillation during follow up, (8) restriction of atrial fibrillation to diagnosis of “Atrial fibrillation” in primary care, (9) exclusion of participants on antiplatelet therapy and (10) exclusion of participants on anticoagulant therapy. Area of each square is proportional to the inverse variance of the estimate.

Supp. Figure 18. Association of baseline atrial fibrillation with the risk of any vascular event. Primary adjustments (1) were for age, BMI, smoking status, sex, baseline diabetes, baseline antihypertensive use, baseline lipid lowering drug (statin) use, baseline anticoagulant usage and baseline antiplatelet usage and interaction between atrial fibrillation and anticoagulant or antiplatelet usage (plotted). Sensitivity analyses were: (2) further adjustment for total cholesterol and HDL cholesterol, (3) further adjustment for total cholesterol, HDL cholesterol and period of blood pressure measurement, (4) exclusion of the first two years of follow up, (5) exclusion of the first four years of follow up, (6) exclusion of individuals who presented with atrial fibrillation three years or more before the baseline blood pressure measurement and have not been diagnosed with atrial fibrillation in the three years prior to the baseline blood pressure measurement, (7) exclusion individuals who developed atrial fibrillation during follow up, (8) restriction of atrial fibrillation to diagnosis of “Atrial fibrillation” in primary care, (9) exclusion of participants on antiplatelet therapy and (10) exclusion of participants on anticoagulant therapy. Area of each square is proportional to the inverse variance of the estimate.

**Web References**

1. Alonso A, Krijthe BP, Aspelund T, Stepas KA, Pencina MJ, Moser CB, Sinner MF, Sotoodehnia N, Fontes JD, Janssens ACJW, Kronmal RA, Magnani JW, Witteman JC, Chamberlain AM, Lubitz SA, Schnabel RB, Agarwal SK, McManus DD, Ellinor PT, Larson MG, Burke GL, Launer LJ, Hofman A, Levy D, Gottdiener JS, Kääb S, Couper D, Harris TB, Soliman EZ, Stricker BHC, Gudnason V, Heckbert SR, Benjamin EJ. Simple risk model predicts incidence of atrial fibrillation in a racially and geographically diverse population: the CHARGE-AF consortium. *J Am Heart Assoc*. 2013;2:e000102–e000102.

2. Conen D, Tedrow UB, Koplan BA, Glynn RJ, Buring JE, Albert CM. Influence of systolic and diastolic blood pressure on the risk of incident atrial fibrillation in women. *Circulation*. 2009;119:2146–2152.

3. Kannel WB, Abbott RD, Savage DD, McNamara PM. Epidemiologic features of chronic atrial fibrillation: the Framingham study. *N Engl J Med*. 1982;306:1018–1022.

4. Krahn AD, Manfreda J, Tate RB, Mathewson FA, Cuddy TE. The natural history of atrial fibrillation: incidence, risk factors, and prognosis in the Manitoba Follow-Up Study. *Am J Med*. 1995;98:476–484.

5. Nyrnes A, Mathiesen EB, Njølstad I, Wilsgaard T, Løchen M-L. Palpitations are predictive of future atrial fibrillation. An 11-year follow-up of 22,815 men and women: the Tromsø Study. *Eur J Prev Cardiol*. 2013;20:729–736.

6. Psaty BM, Manolio TA, Kuller LH, Kronmal RA, Cushman M, Fried LP, White R, Furberg CD, Rautaharju PM. Incidence of and risk factors for atrial fibrillation in older adults. *Circulation*. 1997;96:2455–2461.

7. Roetker NS, Chen LY, Heckbert SR, Nazarian S, Soliman EZ, Bluemke DA, Lima JAC, Alonso A. Relation of systolic, diastolic, and pulse pressures and aortic distensibility with atrial fibrillation (from the Multi-Ethnic Study of Atherosclerosis). *The American Journal of Cardiology*. 2014;114:587–592.

8. Smith JG, Platonov PG, Hedblad B, Engström G, Melander O. Atrial fibrillation in the Malmö Diet and Cancer study: a study of occurrence, risk factors and diagnostic validity. *Eur J Epidemiol*. 2010;25:95–102.

9. Wilhelmsen L, Rosengren A, Lappas G. Hospitalizations for atrial fibrillation in the general male population: morbidity and risk factors. *Journal of internal medicine*. 2001;250:382–389.

10. Bansal N, Fan D, Hsu C-Y, Ordonez JD, Marcus GM, Go AS. Incident atrial fibrillation and risk of end-stage renal disease in adults with chronic kidney disease. *Circulation*. 2013;127:569–574.

11. Watanabe H, Watanabe T, Sasaki S, Nagai K, Roden DM, Aizawa Y. Close bidirectional relationship between chronic kidney disease and atrial fibrillation: the Niigata preventive medicine study. *American Heart Journal*. 2009;158:629–636.

12. Conen D, Chae CU, Glynn RJ, Tedrow UB, Everett BM, Buring JE, Albert CM. Risk of death and cardiovascular events in initially healthy women with new-onset atrial fibrillation. *JAMA : the journal of the American Medical Association*. 2011;305:2080–2087.

13. Chen LY, Sotoodehnia N, Bůžková P, Lopez FL, Yee LM, Heckbert SR, Prineas R, Soliman EZ, Adabag S, Konety S, Folsom AR, Siscovick D, Alonso A. Atrial fibrillation and the risk of sudden cardiac death: the atherosclerosis risk in communities study and cardiovascular health study. *JAMA Internal Medicine*. 2013;173:29–35.

14. Soliman EZ, Safford MM, Muntner P, Khodneva Y, Dawood FZ, Zakai NA, Thacker EL, Judd S, Howard VJ, Howard G, Herrington DM, Cushman M. Atrial fibrillation and the risk of myocardial infarction. *JAMA Internal Medicine*. 2014;174:107–114.

15. Wolf PA, Dawber TR, Thomas HE, Kannel WB. Epidemiologic assessment of chronic atrial fibrillation and risk of stroke: the Framingham study. *Neurology*. 1978;28:973–977.

16. Wolf PA, Abbott RD, Kannel WB. Atrial fibrillation as an independent risk factor for stroke: the Framingham Study. *Stroke*. 1991;22:983–988.

17. Emdin C, Anderson SG, Callender T, Conrad N, Salimi-Khorshidi G, Mohseni H, Woodward M, Rahimi K. Usual blood pressure, peripheral arterial disease, and vascular risk: cohort study of 4.2 million adults. *BMJ*. 2015;351:h4865.

18. Morley KI, Wallace J, Denaxas SC, Hunter RJ, Patel RS, Perel P, Shah AD, Timmis AD, Schilling RJ, Hemingway H. Defining disease phenotypes using national linked electronic health records: a case study of atrial fibrillation. *PLoS ONE*. 2014;9:e110900.

19. Jameson K, Jick S, Hagberg KW, Ambegaonkar B, Giles A, O'Donoghue D. Prevalence and management of chronic kidney disease in primary care patients in the UK. *Int J Clin Pract*. 2014;68:1110–1121.

20. Khan NF, Harrison SE, Rose PW. Validity of diagnostic coding within the General Practice Research Database: a systematic review. *Br J Gen Pract*. 2010;60:e128–36.

21. Johansson S, Wallander MA, Ruigómez A, García Rodríguez LA. Incidence of newly diagnosed heart failure in UK general practice. *Eur J Heart Fail*. 2001;3:225–231.

22. Rapsomaniki E, Timmis A, George J, Pujades-Rodriguez M, Shah AD, Denaxas S, White IR, Caulfield MJ, Deanfield JE, Smeeth L, Williams B, Hingorani A, Hemingway H. Blood pressure and incidence of twelve cardiovascular diseases: lifetime risks, healthy life-years lost, and age-specific associations in 1·25 million people. *The Lancet*. 2014;383:1899–1911.

23. Shah AD, Langenberg C, Rapsomaniki E, Denaxas S, Pujades-Rodriguez M, Gale CP, Deanfield J, Smeeth L, Timmis A, Hemingway H. Type 2 diabetes and incidence of cardiovascular diseases: a cohort study in 1·9 million people. *Lancet Diabetes Endocrinol*. 2014;0.

24. Herrett E, Thomas SL, Schoonen WM, Smeeth L, Hall AJ. Validation and validity of diagnoses in the General Practice Research Database: a systematic review. *Br J Clin Pharmacol*. 2010;69:4–14.
